# Engineering *Saccharomyces cerevisiae* for geranylgeraniol overproduction by combinatorial design

**DOI:** 10.1038/s41598-017-15005-4

**Published:** 2017-11-08

**Authors:** Tian-Qing Song, Ming-Zhu Ding, Fang Zhai, Duo Liu, Hong Liu, Wen-Hai Xiao, Ying-Jin Yuan

**Affiliations:** 1 Key Laboratory of Systems Bioengineering (Ministry of Education), Tianjin University, Tianjin, 300072 P.R. China; 2 SynBio Research Platform, Collaborative Innovation Center of Chemical Science and Engineering (Tianjin), School of Chemical Engineering and Technology, Tianjin University, Tianjin, 300072 P.R. China

## Abstract

Combinatorial design is an effective strategy to acquire the optimal solution in complex systems. In this study, the combined effects of pathway combination, promoters’ strength fine-tuning, copy numbers and integration locus variations caused by δ-integration were explored in *Saccharomyces cerevisiae* using geranylgeraniol (GGOH) production as an example. Two GGOH biosynthetic pathway branches were constructed. In branch 1, GGOH was converted from isopentenyl pyrophosphate (IPP) and farnesyl diphosphate (FPP). In branch 2, GGOH was derived directly from IPP and dimethylallyl pyrophosphate (DMAPP). Regulated by 10 combinations of 11 diverse promoters, a fusion gene *BTS1-ERG20*, a heterologous geranylgeranyl diphosphate synthase from *Sulfolobus acidocaldarius* (*GGPPSsa*) and an endogenous N-terminal truncated gene 3-hydroxyl-3-methylglutaryl-CoA reductase isoenzyme 1 (*tHMGR*), were incorporated into yeast by δ-integration, leading to a series of GGOH producing strains with yields ranging from 18.45 mg/L to 161.82 mg/L. The yield was further increased to 437.52 mg/L by optimizing the fermentation medium. Consequently, the GGOH yield reached 1315.44 mg/L in a 5-L fermenter under carbon restriction strategy. Our study not only opens large opportunities for downstream diterpenes overproductions, but also demonstrates that pathway optimization based on combinatorial design is a promising strategy to engineer microbes for overproducing natural products with complex structure.

## Introduction

(E, E, E)-Geranylgeraniol (GGOH), a kind of ingredient for perfumes, can also be used to synthesize vitamins A and E^1,2^. In *Saccharomyces cerevisiae*, GGOH is the dephosphorylated derivative of geranylgeranyl pyrophosphate (GGPP) by its endogenous phosphatases. In general, GGOH production is too low to be detected in the wild strains. Since GGPP is the direct substrate for diterpenoids synthesis in microorganisms, developing GGPP overproduction platform in yeast would open many opportunities for other high value compounds biosynthesis^3^. In the meanwhile, taken the difficulty of GGPP detection into consideration, GGOH can be utilized as another direct reporter for GGPP overproduction except lycopene and β-carotene. Recently, researchers have focused on the overproduction of GGOH in microbial hosts through metabolic engineering and synthetic biology strategies. Tokuhiro *et al*. achieved a titer of 228.8 mg/L GGOH in an engineered diploid prototrophic *S. cerevisiae* through overexpression of *BTS1-DPP1* fusion gene, *BTS1-ERG20* fusion gene, and the *HMG1* genes^4^. Ohto *et al*. obtained a yield of 138.8 mg/L GGOH in a 5-L jar by combined effects of co-expression of *HMG1* and the *BTS1-ERG20* fusion and culture conditions optimization^5^. However, all above efforts just focused on fine-tuning endogenous GGPP biosynthesis pathway using IPP and FPP as precursors, in which the FPP is also an intermediate for ergosterol accumulation. It was also reported some heterologous *GGPPS* could directly catalyze IPP/DMAPP into GGPP, which can efficiently avoid the competition from ergosterol biosynthesis. Thus, co-overexpression and combinatorial fine-tuning of the two pathways would be crucial for GGPP overproduction as well as GGOH accumulation.

Combinatorial design has been widely used in synthetic biology and metabolic engineering areas for microbial production of diverse chemicals^6–8^. Firstly, multiple pathways with same or similar function can be combinatorially assembled to enhance target products output, Lv *et al*. succeeded in boosting isoprene synthesis in *S. cerevisiae* by dual metabolic engineering of cytoplasmic and mitochondrial acetyl-CoA utilization^9^. Secondly, as promoters are the key regulators of gene expression, different strength of promoters facilitated the fine-tuning of gene expression levels^10–13^. Kim *et al*. reported enhanced production of 2,3-butanediol was achieved in engineered *S. cerevisiae* through fine-tuning of pyruvate decarboxylase and NADH oxidase activities^14^. The manipulation of copy numbers and insertion sites of targeted genes are also an effective solution to realize diverse expression levels^15,16^. The δ-integration has been widely used for the integration of heterologous genes and the construction of biosynthetic pathways and can simultaneously achieve significant disturbances in both gene copy numbers and insertion locations^17,18^. The delta-sequences are yeast retrotransposon Ty1 long terminal repeats (LTR) sequences, and they have more than 400 copies distributed throughout the yeast genome. Unlike other single copy sites, δ-site integration has the potential to achieve multiple stable integrations at a time through yeast homologous recombination, and the integration efficiency is related to some factors, such as the size and the number of the integration fragments. Semkiv *et al*. constructed a vector for multicopy δ-integration in *S. cerevisiae* using a modified selective marker and a reporter gene *PHO8*. Their system provided integration of 3–10 copies of the module in the genome of *S. cerevisiae* ^19^. Therefore, combining all above aspects could efficiently improve the output of desired products in a more systematic way.

In the present study, three main modules involved in GGOH production, endogenous gene *tHMGR*, a fusion gene *BTS1-ERG20*, and a heterologous gene *GGPPSsa* from *S. acidocaldarius* under the control of 10 combinations of 11 various promoters were introduced into yeast by δ-integration, generating a series of GGOH producing strains, in which the yields varied from 18.45 mg/L to 161.82 mg/L. The effects of integration sites and copy numbers on GGOH production were also investigated. Finally, a titer of 1315.44 mg/L GGOH was achieved in a 5-L bioreactor based on carbon restriction strategy. Our research provides an efficient platform for GGOH and GGPP producing pool, which can largely promote downstream terpenes biosynthesis. Our study also sets a reference for microbial overproduction of desired chemicals and pharmaceuticals through combinatorial pathways design.

## Results and Discussion

### Pathway combination for GGOH biosynthesis

**Figure 1:**
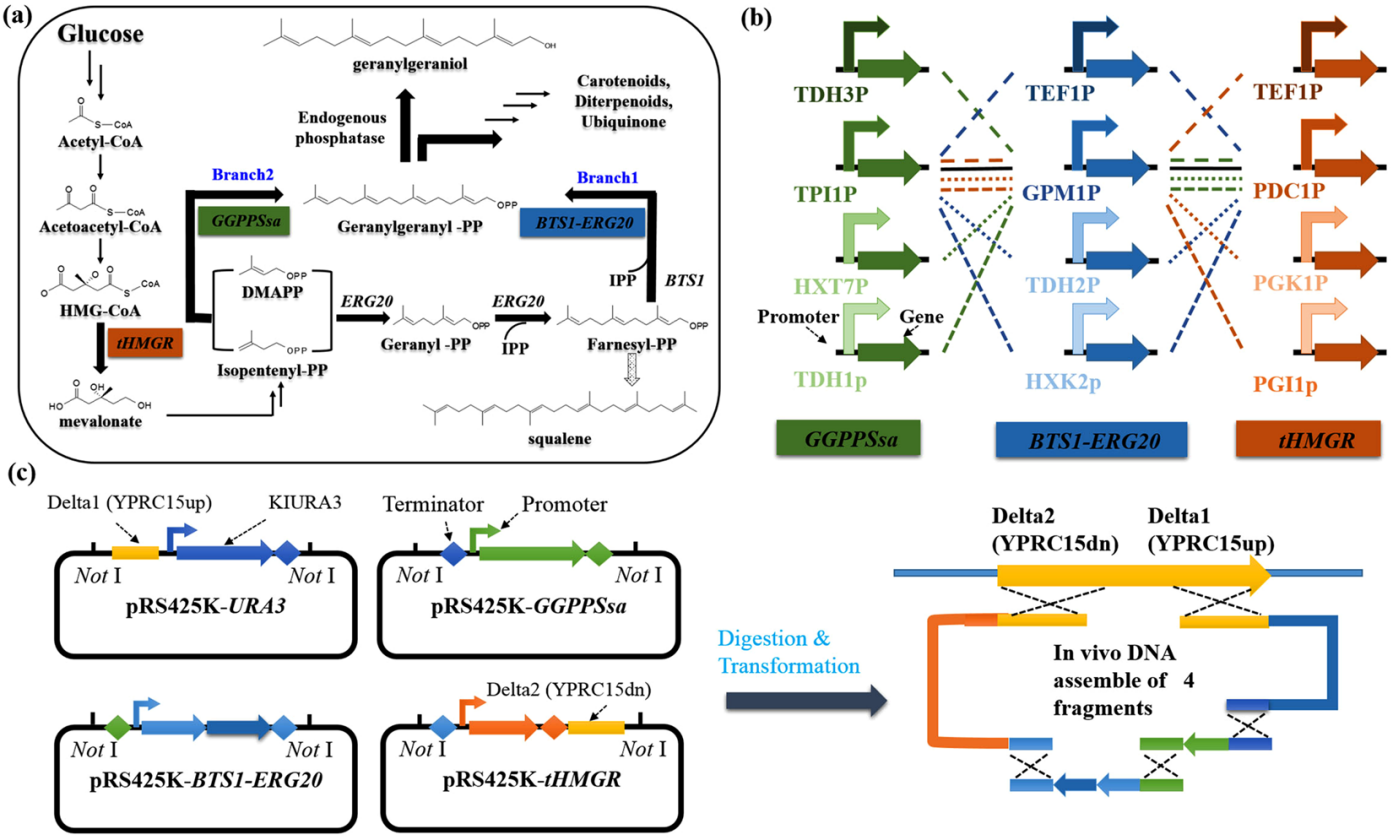
Paradigm of combinatorial design for GGOH biosynthesis in *S. cerevisiae*. (**a**) Pathway combination for GGOH production. Key enzymes are *tHMGR*, fusion protein *BTS1-ERG20* and *GGPPSsa*. (**b**) Combinatorial design of GGOH biosynthetic modules with different promoter strength. Each color and line type represented an independent combination. (**c**) Schematic diagram for one-step δ-integration.

The pathway combination for the construction of GGOH biosynthetic pathway in yeast was described in Fig. 1a. To compete with hidden pathways that may inhibit the production of our desired product and to increase the flux from IPP to GGPP, both of the branch 1 and branch 2 for GGPP production were combinatorially constructed by co-overexpression of the fusion gene *BTS1-ERG20* and the exogenous *GGPPSsa* gene (Fig. 1a). Another major regulatory control point of the MVA pathway is HMG-CoA reductase (*HMGR*). Early studies by Donald *et al*.^20^ and Ohto *et al*.^5^ showed that over-expression of the catalytic domain of HMGR could lead to improved productions of isoprenoids. We thus integrated the *tHMGR* cassette into the genome in this study as well.

Since YPRC15 locus was reported with higher gene expression level among 20 different integration sites in *S. cerevisiae* genome^21^, we transformed the three modules (*GGPPSsa*, *BTS1-ERG20*, *tHMGR*) into the YPRC15 locus in the genome of BY4742, obtaining strain SyBE_Sc01010369. As shown in Supplementary Fig. S1, after 72 h in shake flask cultivation, a distinct peak with retention time 13.44 min was detected in strain SyBE_Sc01010369, which was identified as GGOH based on retention time and mass spectrum (69, 93, and 119 m/z), indicating that the biosynthesis pathway of GGOH was successfully constructed in yeast. In addition, the titer of this strain we firstly constructed was only 24.93 mg/L.

### The diversity of GGOH production obtained by promoters’ combination and δ-integration

**Figure 2:**
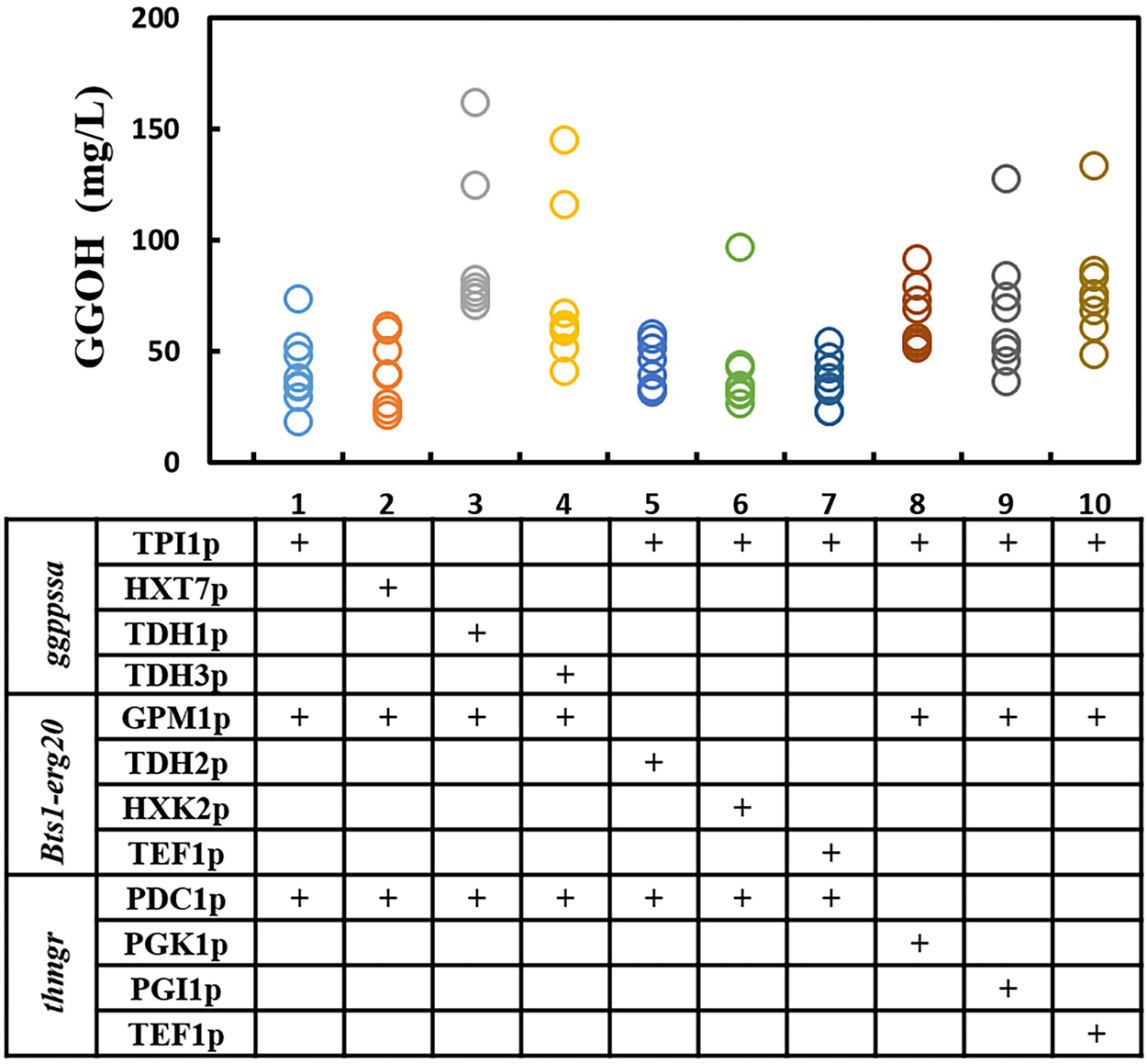
The diversity of GGOH production obtained by promoters’ combination and δ-integration. 8 transforments of each combination were investigated. Each circle represented an independent transformant.

Since there are two constructed pathway branches for co-producing GGOH (Fig. 1), the relative low yield of GGOH production at present may be due to unbalanced expression levels of the three modules. Systematic and combinatorial fine-tuning of the interactive modules would be an efficient solution to obtain balanced metabolic flux towards GGOH. This fine-tuning can be obtained through a selection of promoters, integration sites and copy numbers using δ-integration. According to the work of Zhao *et al*.^11^, 11 promoters with different strength were chosen for strains construction. The strength of all the promoters involved in this study were characterized by RFP as shown in Supplementary Fig. S2. In present study, as shown in Fig. 1b and Fig. 1c, one-step genomic δ-integration of the three modules with 10 combinations of 11 diverse promoters was performed in BY4742 strain. In the meanwhile, eight transformants of each constructed combination were chosen and cultivated, generating a wide dynamic library of GGOH-producing strains (obtaining strains SyBE_Sc00011201 to SyBE_Sc00011280) (Table 1) in which the yields varied from 18.43 mg/L to 161.82 mg/L (Fig. 2). Previous study by Yuan and Ching^22^ obtained a amorpha-4, 11-diene yield range approximately from 5 mg/L to 64 mg/L by the δ-integration of the MVA pathway genes together with carotenoid-based screening approach. The consistency in production library between our study and other group just support the efficiency of one-step δ-integration in manipulation of multiple modules. Based on this combinatorial design, a highest GGOH producing strain named SyBE_Sc00011224 was generated for further bioprocess optimization.

**Table 1:** Strains used in this study.

Name	Description	Reference
BY4742	*MATα his3Δ1 leu2Δ0 lys2Δ0 ura3Δ0*	Baker Brachmann, C. *et al*. 1998^34^
SyBE_Sc01010369	YPRC15:: TPI1p-*GGPPSsa*-GPM1p-*BTS1-ERG20*-PDC1p-*tHMGR*	This study
SyBE_Sc00011201 SyBE_Sc00011202 SyBE_Sc00011203 SyBE_Sc00011204 SyBE_Sc00011205 SyBE_Sc00011206 SyBE_Sc00011207 SyBE_Sc00011208	Combination 1: Delta:: TPI1p-*GGPPSsa*-GPM1p-*BTS1-ERG20*-PDC1p-*tHMGR*	This study
SyBE_Sc00011209 SyBE_Sc00011210 SyBE_Sc00011211 SyBE_Sc00011212 SyBE_Sc00011213 SyBE_Sc00011214 SyBE_Sc00011215 SyBE_Sc00011216	Combination 2: Delta:: HXT7p-*GGPPSsa*-GPM1p-*BTS1-ERG20*-PDC1p-*tHMGR*	This study
SyBE_Sc00011217 SyBE_Sc00011218 SyBE_Sc00011219 SyBE_Sc00011220 SyBE_Sc00011221 SyBE_Sc00011222 SyBE_Sc00011223 SyBE_Sc00011224	Combination 3: Delta:: TDH1p-*GGPPSsa*-GPM1p-*BTS1-ERG20*-PDC1p-*tHMGR*	This study
SyBE_Sc00011225 SyBE_Sc00011226 SyBE_Sc00011227 SyBE_Sc00011228 SyBE_Sc00011229 SyBE_Sc00011230 SyBE_Sc00011231 SyBE_Sc00011232	Combination 4: Delta:: TDH3p-*GGPPSsa*-GPM1p-*BTS1-ERG20*-PDC1p-*tHMGR*	This study
SyBE_Sc00011233 SyBE_Sc00011234 SyBE_Sc00011235 SyBE_Sc00011236 SyBE_Sc00011237 SyBE_Sc00011238 SyBE_Sc00011239 SyBE_Sc00011240	Combination 5: Delta:: TPI1p-*GGPPSsa*-TDH2p-*BTS1-ERG20*-PDC1p-*tHMGR*	This study
SyBE_Sc00011241 SyBE_Sc00011242 SyBE_Sc00011243 SyBE_Sc00011244 SyBE_Sc00011245 SyBE_Sc00011246 SyBE_Sc00011247 SyBE_Sc00011248	Combination 6: Delta:: TPI1p-*GGPPSsa*-HXK2p-*BTS1-ERG20*-PDC1p-*tHMGR*	This study
SyBE_Sc00011249 SyBE_Sc00011250 SyBE_Sc00011251 SyBE_Sc00011252 SyBE_Sc00011253 SyBE_Sc00011254 SyBE_Sc00011255 SyBE_Sc00011256	Combination 7: Delta:: TPI1p-*GGPPSsa*-TEF1p-*BTS1-ERG20*-PDC1p-*tHMGR*	This study
SyBE_Sc00011257 SyBE_Sc00011258 SyBE_Sc00011259 SyBE_Sc00011260 SyBE_Sc00011261 SyBE_Sc00011262 SyBE_Sc00011263 SyBE_Sc00011264	Combination 8: Delta:: TPI1p-*GGPPSsa*-GPM1p-*BTS1-ERG20*-PGK1p-*tHMGR*	This study
SyBE_Sc00011265 SyBE_Sc00011266 SyBE_Sc00011267 SyBE_Sc00011268 SyBE_Sc00011269 SyBE_Sc00011270 SyBE_Sc00011271 SyBE_Sc00011272	Combination 9: Delta:: TPI1p-*GGPPSsa*-GPM1p-*BTS1-ERG20*-PGI1p-*tHMGR*	This study
SyBE_Sc00011273 SyBE_Sc00011274 SyBE_Sc00011275 SyBE_Sc00011276 SyBE_Sc00011277 SyBE_Sc00011278 SyBE_Sc00011279 SyBE_Sc00011280	Combination 10: Delta:: TPI1p-*GGPPSsa*-GPM1p-*BTS1-ERG20*-TEF1p-*tHMGR*	This study

As described in Fig. 2, it was also found that the GGOH yields were significantly different when changing the promoters of the *GGPPSsa* (combinations 1, 2, 3 and 4), indicating that fine-tuning of the *GGPPSsa* gene might be more favorable for higher GGOH production. However, the promoter of the *GGPPSsa* with the highest GGOH production (TDHIp) was not the strongest one (TDH3p) among the four promoters tested. *GGPPSsa* could catalyze IPP/DMAPP, GPP and FPP to GGPP, but the enzyme activity for these substrates decreased sequentially^23^. We deduced that the fluctuations of the GGOH production caused by fine-tuning of the expression level of *GGPPSsa* were due to the complicate and non-linear catalytic characteristics and the wide compatibility with substrates as well as its synergistic effect with the other pathway branch in this study. This assumption was further proved by the modest but relatively apparent fluctuation of GGOH production caused by regulating *BTS1-ERG20* in branch 1 (combinations 1, 5, 6 and 7). Similar results were found when promoters’ strength of *tHMGR* changed (combinations 1, 8, 9 and 10). We believed that fine-tuning the promoter strength of each single gene or pathway branch simultaneously led to balanced metabolic flux and thereby influenced the overall production of desired product in our study.

**Figure 3:**
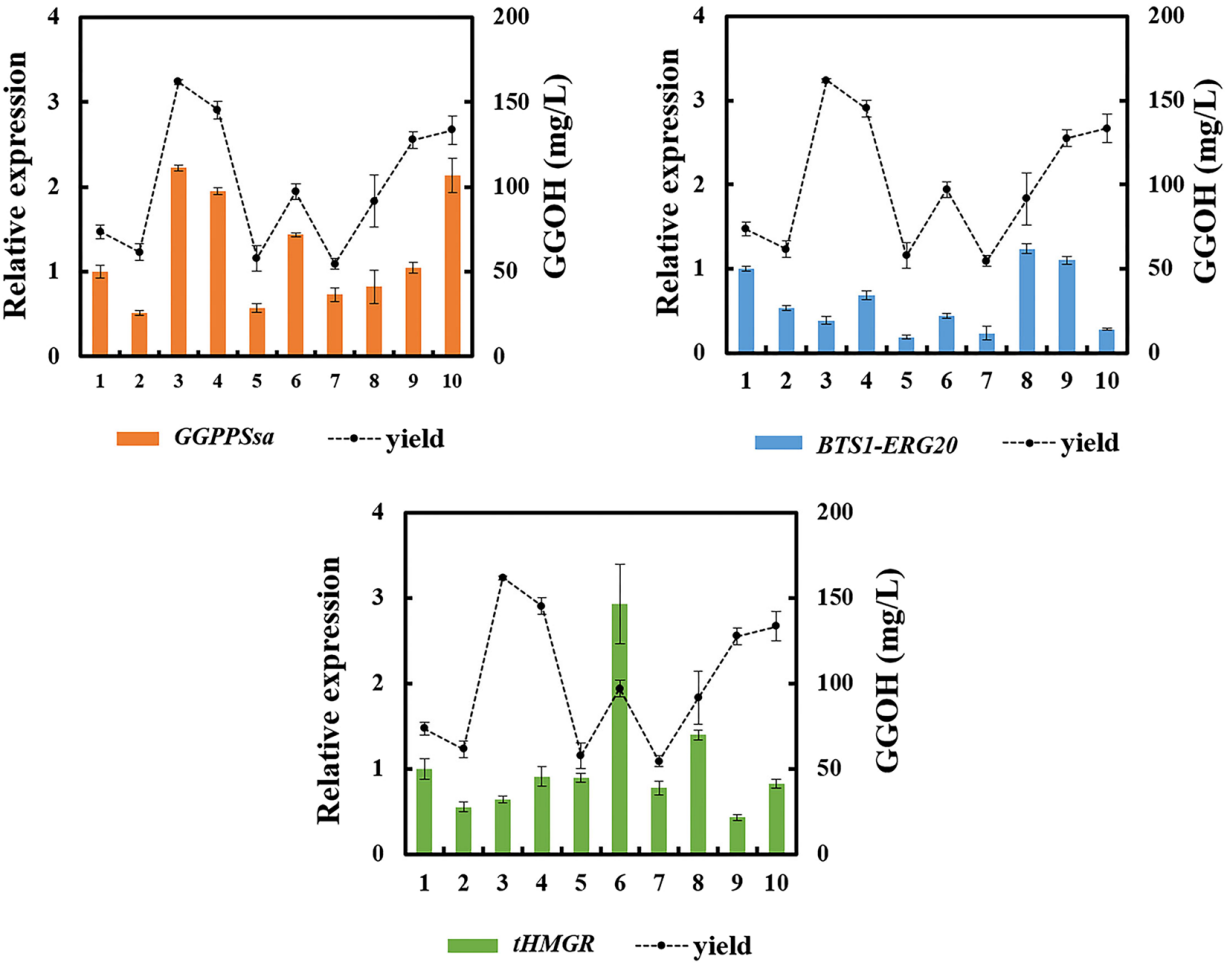
Transcriptional analysis of *tHMGR*, *BTS1-ERG20*, *GGPPSsa* in transformants with highest GGOH yields from each combination. The expression level of each gene was determined by real time PCR. The yield data was calculated from triplicates.

Furthermore, the gene expression levels of *tHMGR*, *BTS1-ERG20*, and *GGPPSsa* in strains with the highest GGOH production in each combination were also investigated by RT-PCR for better understanding of the underlying mechanisms that would affect the GGOH production (Fig. 3). It was shown that the diversity of expression levels for each module was realized by one-step δ-integration and the transcriptional levels of *GGPPSsa* were positively correlated with the GGOH yields, indicating that *GGPPSsa* played a dominant role in the GGOH production pathway. The *GGPPSsa* can directly use the IPP and DMAPP, which had higher priority and stronger competitiveness on the substrates supply compared to *BTS1-ERG20*, leading to more metabolic flux to GGPP than FPP. Furthermore, previous study showed *GGPPSsa* not only had IPP catalytic activity, but also possessed GPP and FPP catalytic activity^24^. In addition, the K_m_ value for IPP and FPP of *GGPPSsa* was 0.361 μM and 1.8 μM, which was lower than the corresponding K_m_ value of *BTS1* as 0.8 μM and 3.2 μM^25^, which also indicated that *GGPPSsa* had a better affinity for these allylic diphosphates, and thus played a decisive role.

**Figure 4:**
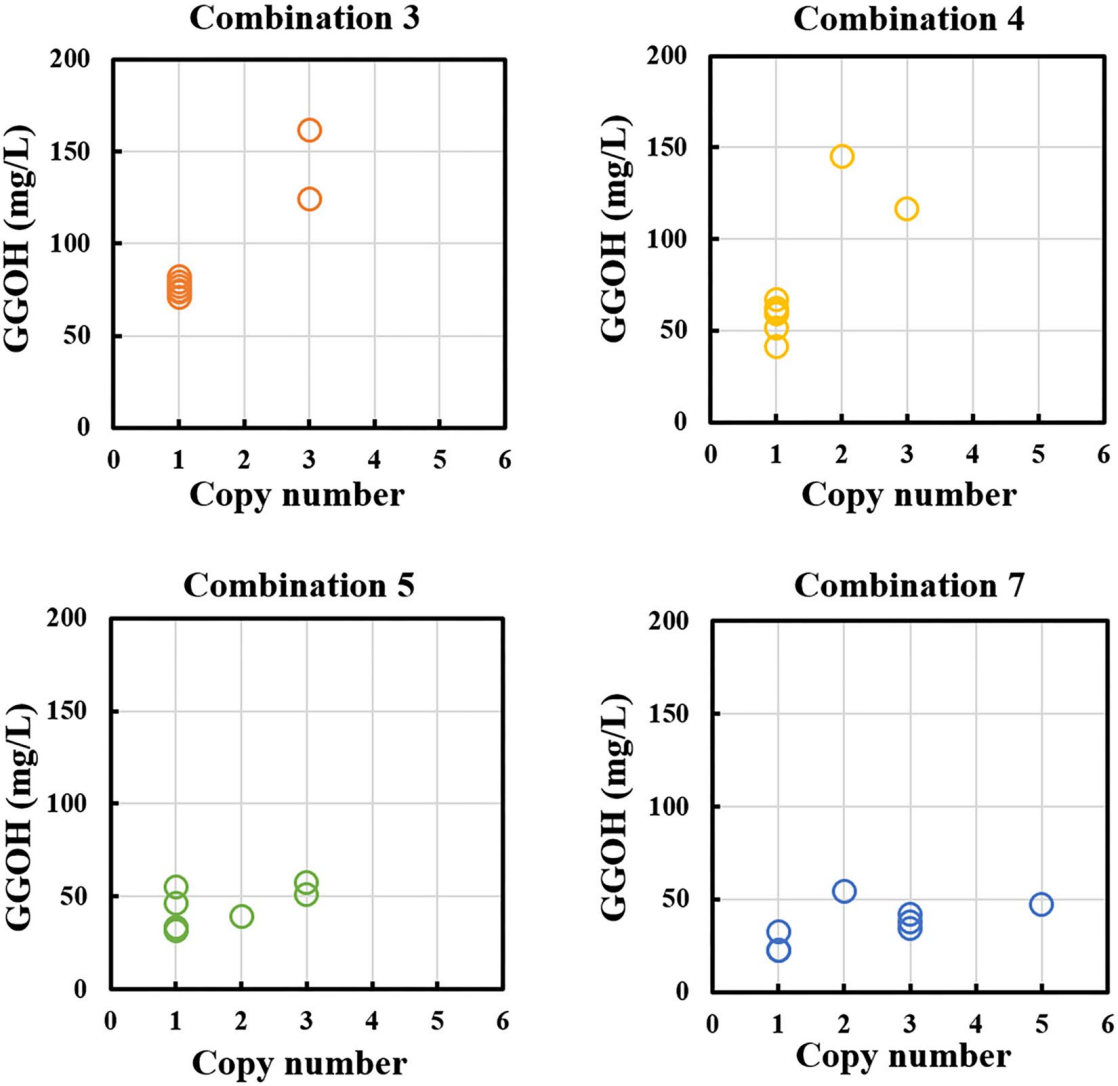
The correlation between copy numbers and GGOH yields. All the samples were from combinations 3, 4, 5, and 7.

In addition, the δ-integration could lead to multiple copies integrating in different sites, and thus improve the integration efficiency. Regardless of the difference of viability between parent strains, the reason for the production variation between 8 transformants in each combination was thought to be the different copy numbers and integration sites caused by δ-integration. Firstly, in order to verify the effect of copy number on GGOH yield, we selected combinations 3, 4 with high-yield, and combinations 5, 7 with low-yield to verify the effects of the integration copy number on GGOH production in each transformant in these combinations by qPCR. In all the 32 transformants, 20, 3, 8 and 1 transformants possessed 1, 2, 3 and 5 copy integrations, respectively, which was consistent with the previous study that the use of auxotrophic selection marker often resulted in single integration into yeast chromosome^26^. Further confirmation of multiple-copy site integration and increasing the size of the screening library are likely to obtain strains with higher copies in future. Besides, the copy number could be further increased using recently developed antibiotic selection markers^19,27^, and CRISPR-mediated δ-integration^28^. As shown in Fig. 4, taken the yields into consideration, there was a nonlinear relationship between yields and copy numbers. Taking combination 7 as an example, the GGOH yield in strain with 2 copies was 54.54 mg/L, while the yield was only 47.57 mg/L in strain with 5 copies. In addition, there were also differences in yields among strains with the same copy number and promoter combination. For example, the yields of the 1 copy integration in combination 5 ranged from 31.95 mg/L to 55.32 mg/L. Recent study has also shown that position effects accounted for increased variability in gene expression levels^16^. Thus, expression level differences among these strains with one copy δ-integration may be due to the position effect, even though its inherent reason is not clear yet. Thus, we also have tried to figure out the differences in the integration sites, 10 specific strains were selected from combinations 3, 4, 9 and 10 (see Supplementary Table S1) with diverse GGOH production, and tested by PCR using one certain forward primer annealing to the end of the integrated module with reverse primers bonding to the downstream of the delta sequence on the chromosome (see Supplementary Fig. S3a). 25 δ-sites gained from NCBI Nucleotide BLAST (https://blast.ncbi.nlm.nih.gov/Blast.cgi) with more than 90% sequence similarity, were chosen for verification by PCR, and 6 δ-sites were confirmed with fragments integration. The result showed that the integration sites of delta-integration were diversified, but the correlation between integration sites and GGOH productions was not apparent (see Supplementary Fig. S3b and Supplementary Table S1). In summary, our results demonstrated that the variety by δ-integration would facilitate a large range for productions of target compounds. The δ-integration combined with fine-tuning of promoters’ strength provided a comprehensive and efficient solution for obtaining high-producing strains through synthetic biology and metabolic engineering. Nevertheless, the fact that different integration sites gave rise to different production levels does not mean that all production levels can be explained through copy numbers and integration sites, which needs further illustration in future work.

### GGOH production promoted by medium optimization and fed batch fermentation

**Figure 5:**
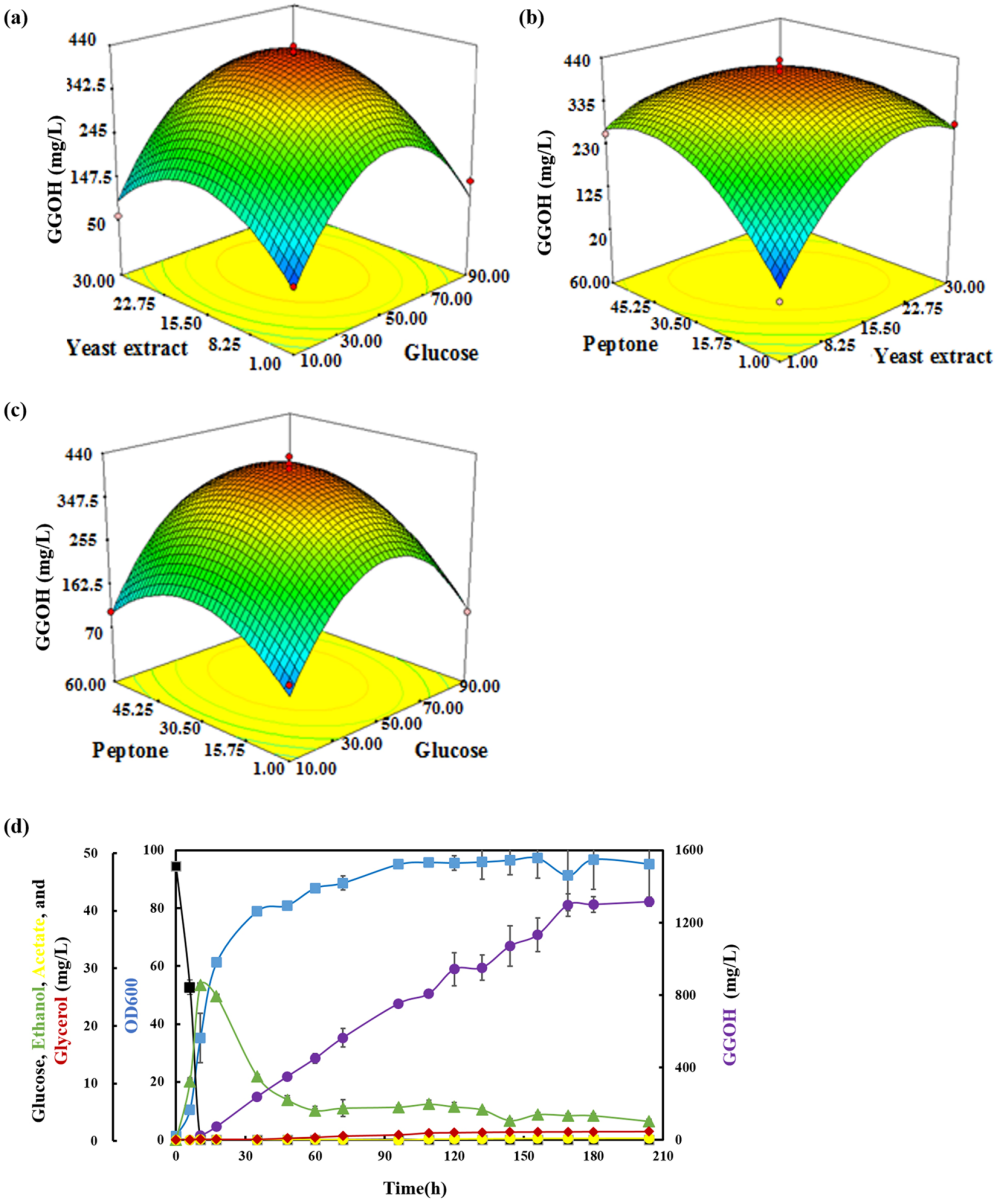
Medium optimization and fed-batch fermentation. (**a**) Response surface methodology for medium optimization. (**b**) 5-L Fed-batch fermentation. Line in dark, green, yellow, red, blue and purple represented glucose, ethanol, acetate, glycerol, OD_600_ and GGOH, respectively. All the data points were calculated from duplicates.

In order to determine the optimal fermentation medium for further bioreactor production of GGOH, systematic optimization was performed using the highest GGOH yield strain in the combination 3, named SyBE_Sc00011224. Firstly, the 2-level fractional factorial design was utilized to investigate the main effects and interactions of glucose, yeast extract, peptone, MgSO_4_ and KH_2_PO_4_ (see Supplementary Table S2). The result (see Supplementary Fig. S4) showed that the composition of glucose, yeast extract and peptone significantly influenced GGOH production. Then, the three critical factors were further determined by applying Response Surface Methodology (RSM) with a Box-Behnken design (see Supplementary Table S3) whereas concentrations of KH_2_PO_4_ and MgSO_4_ were fixed on the constant levels. The experimental results were analyzed statistically using ANOVA. The 3D surface plots of every two factors were shown in Fig. 5a,b,and c, and the final equation in terms of coded factors was presented in Equation S1. Based on the equation, optimal solution of the concentration was 52.9 g/L glucose, 16.4 g/L yeast extract and 32.8 g/L peptone_,_ and the maximum titer of GGOH was predicted to reach 420.00 mg/L. The verification experiment was done with the predicted concentration of medium and the actual GGOH production was 437.52 ± 30.18 mg/L, which matched the prediction very well. The good agreement between the prediction and experimental results verified the validity of the model. This titer in the optimized fermentation medium was increased by 1.75-fold than that in the traditional YPD medium.

Statistical optimization is an important step for overproduction of the desired product and involves a number of physiochemistry parameters^29,30^. Microbes that are engineered to target metabolites always have different requirements for nutrients from the original ones due to the shift of metabolic flux caused by introduction of heterologous pathways. Through rational optimization, the most favorable and well-balanced compositions of nutrients can be easily obtained which facilitates the maximum yield of the desired product^31,32^. The enhancement of cell growth and GGOH production through statistical optimization in this study illustrated that there would be still huge potential for shifts in metabolism by regulating medium components. It could be speculated that combinatorial integrative design provided a high possibility for increasing the yield of target product by RSM.

In order to fully explore the GGOH producing ability in the engineered strains, fed-batch fermentation of the highest GGOH accumulating strain (437.52 ± 30.18 mg/L in strain SyBE_00011224) was carried out in a 5-L bioreactor (Fig. 5d) with liquid-liquid two-phase fermentation using 20% (v/v) dodecane as overlay. The organic solvent was introduced to prevent air stripping of secreted GGOH from the fermentation medium. In defined media with controlled glucose feeding, GGOH production of the strain SyBE_Sc00011224 increased to 1315.44 mg/L within 200 h. In addition, the biomass increased dramatically during the first 35 h when the yield of GGOH reached 238.49 mg/L. Then, the strain kept a relatively stable growth rate during the next 165 h, and the titer increased significantly from 238.49 mg/L to 1315.44 mg/L. During the whole process of fermentation, GGOH maintained a stable growth rate, which proved that there was great potential to further increase production. However, it was also noticed that the fermentation time was quite long. As recent study in process optimization has demonstrated great potential in isoprene overproduction (up to 24.0 g/L)^33^, we believe that GGOH and related other terpenoids production by our engineered strain would be further improved by continuous efforts in metabolic engineering, synthetic biology and fermentation optimization.

## Conclusion

In this study, combinatorial design was carried out to optimize the GGOH titer in engineered *S. cerevisiae*. A GGOH overproducing *S. cerevisiae* with the titer of 1315.44 mg/L in the 5-L bioreactor was obtained by constructing two GGOH biosynthetic pathway branches, fine-tuning the promoters’ strength of key genes and optimizing the fermentation conditions. This study provides a possibility for obtaining high-production strains by combinatorial design. Thus, it would be a helpful strategy for constructing microbes by combinatorial design to overproduce terpenoid and other natural products. The improvement of the GGOH titer in *S. cerevisiae* chassis constructs a sufficient GGPP supply pool and would eventually offer a great opportunity for the biosynthesis of taxol precursors overproduction, such as taxadiene and oxygenated taxanes in microbes.

## Methods

### Strains and plasmids

All the plasmids were constructed in *E. coli* DH5a, and all the GGOH producing strains were constructed in the yeast strain BY4742, a derivative of S288C^34^, was obtained from EUROSCARF and used as the parent strain for all yeast strains. Plasmids extraction was purchased from BEIJING Biomed Co., Ltd. The yeast expression plasmid pRS425 was purchased from Addgene (American) and the ampicillin resistant gene of the plasmid was substituted by kanamycin resistant gene to construct pRS425K. The yeast strains constructed in this study were listed in Table 1.

### Combinatorial design and plasmid construction

The GGOH biosynthetic pathway branches were constructed by combinatorial design (Fig. 1a). The GGOH producing strains were engineered followed the illustration shown in Fig. 1b and c. For the production of GGOH, yeast endogenous gene *tHMGR, ERG20* and *BTS1* were amplified by PCR from the genomic DNA of the strain BY4742. The fusion enzyme encoding gene *BTS1-ERG20* was constructed using overlap extension PCR (OE-PCR) according to the method described by Zhou *et al*.^35^. The codon optimization and complete synthesis of *GGPPS* gene from *S. acidocaldarius (GGPPSsa)* were performed by AuGCT Company (China). Promoters with different strength used in this study were shown in Fig. 1b and Supplementary Table S4, and were all amplified by PCR from the genomic DNA of the strain BY4741. All the cassettes built in this study were listed in Supplementary Table S4, and primers used were listed in Supplementary Table S5. In order to integrate the pathway modules into the YPRC15 locus, four cassettes YPRC15up-*ura3*-CYC1t, CYC1t-TPI1p-*GGPPSsa*-TEF1t, TEF1t-GPM1p-*BTS1-ERG20*-TPI1t, and TPI1t-PDC1p-*tHMGR*-FBA1t-YPRC15dn were built in the vector pRS425K separately with their terminator acting as homologous fragment to each other. Specifically, for the pRS425K- YPRC15up-*ura3*-CYC1t cassette, the YPRC15 locus upstream 600 bp homologous fragment YPRC15up, the URA3 marker gene from *Kluyveromyces lactis* and the CYC1 terminator were constructed by OE-PCR. The resulting fragments were digested with *Not*I, and introduced into the corresponding sites of vector pRS425K to form pRS425K-YPRC15up-*ura3*-CYC1t. For pRS425K-CYC1t-TPI1p-*GGPPSsa*-TEF1t cassette, the empty cassette pRS425K-CYC1t-TPI1p-TEF1t was first constructed by OE-PCR, consisting of upstream homologous fragment CYC1 terminator, promoter TPI1p, and downstream terminator TEF1 into the plasmid pRS425K. Then, the gene *GGPPSsa* was introduced into the empty cassettes constructed above to create pRS425K-CYC1t-TPI1p-*GGPPSsa*-TEF1t cassettes using the Golden Gate method^36^. The pRS425K-TEF1t-GPM1p-*BTS1-ERG20*-TPI1t cassette was constructed following the similar steps as pRS425K-*GGPPSsa* cassette. For pRS425K-TPI1t-PDC1p-*tHMGR*-FBA1t-YPRC15dn cassette, YPRC15dn was introduced into the end of the second terminator as the downstream homologous region with yeast chromosome. Correct constructions of the cassettes were confirmed by diagnostic PCR using primers annealing to the end of the promoter and the beginning of the terminator. In each case, the correct insertion was confirmed by vector insert sequencing. Then, the upstream (YPRC15up) and downstream (YPRC15dn) homologous modules were replaced by delta-site upstream homologous fragment delta1 and downstream homologous fragment delta 2 for δ-integration. Besides, promoters with different strengths were introduced into corresponding cassettes following the same method above except for the TEF1p regulated cassettes. Since there was a *Bsa*I restriction site in the nucleotide sequence of TEF1p, *Pme*I site was introduced into the TEF1p end instead of *Bsa*I and yeast homologous recombination was applied to *in vivo* assembling the *Pme*I cleaved plasmid and the gene.

The expression levels of the 11 promoters used in this study were tested by RFP. Different promoters were digested by *Bgl*II */Xba*I and were introduced into the corresponding site of vector pRS425K-KAX-GFP-Leu, which was integrated at the KAX site of the BY4742 strain.

### Yeast transformation, verification and cultivation

The target pathways were constructed by DNA assembler^37^. The cassettes described above were digested with corresponding restriction enzymes, and the URA3 maker fragment and the gene fragments related to the GGOH pathway were co-transformed into yeast after gel purification as designed in Fig. 1c. Yeast was transformed using the LiAc/SS carrier DNA/PEG method followed by selection on SC-drop agar plates (0.2% amino acid mixture, 0.67% yeast nitrogen base without amino acid, 2% glucose, 1.8% agar) without supplementation of uracil. After incubating at 30 °C for 36 h, 12 colonies were selected to be cultivated in SC-drop medium at 220 rpm for 20 h before the genomes were extracted. The genomes of the 12 colonies were extracted and verified by PCR with primers listed in Supplementary Table S5 and the genome of the BY4742 chassis was used as negative control. For GGOH production, seed was firstly inoculated from SC-drop agar plate into 20 mL culture tubes containing 5 mL SD medium and cultivated at 30 °C, 220 rpm for 20 h to the exponential phase (OD_600_≈5.0). And then aliquots were diluted to an initial OD_600_ of 0.2 in 5 mL of SD medium and cultivated at 30 °C, 220 rpm for 12 h until OD_600_ reached about 5.0. Aliquots were diluted to an initial OD_600_ of 0.1 in 50 mL of YPD medium (1% yeast extract, 2% peptone, 2% glucose) in 250 mL flasks and were cultivated at 30 °C, 220 rpm. Dodecane was added aseptically to 5% (v/v) of the culture at 10 h to minimize the loss of GGOH. And the organic layer was harvested for GGOH analysis after 70 h by centrifugation of the fermentation broth at 11,000 rpm for 10 min.

### Fed batch Fermentation for GGOH production

The experiment of fractional factorial designs and response surface methodology (RSM) were calculated using Design Expert Software, Stat-Ease, Inc. 1.5 g/L citric acid which served as chelating agent was introduced into the medium as MgSO_4_ and KH_2_PO_4_ were added. The fermentation processes at shake-flask level were performed as described above.

A 5-L bioreactor (BLBIO-5GJG-2, Shanghai, China) was used for this study. Firstly, cells from a single colony of SyBE_00011224 on the SC-drop agar plate was cultivated in a 100 mL flask containing 20 mL SC-drop medium at 30 °C, 200 rpm for 20 h, and then aliquots were diluted to an initial OD_600_ of 0.2 in 200 mL of SD medium and cultivated for 10 h until OD_600_ reached about 5.0. Secondly, 200 mL seed culture was transferred into the bioreactor containing 1.8 L fermentation medium (5% glucose, 1% yeast extract, 3% peptone, 0.8% KH_2_PO_4_, and 0.6% MgSO_4_). Oxygen was supplied by filtered air at 1 vvm. The agitation was 350 rpm. The temperature was maintained at 30 °C. The pH of the culture was controlled at 5.8 using 10 M ammonia water. 20% (v/v) dodecane was added aseptically into the culture at 6 h of the fermentation to start the biphasic liquid-liquid fermentation. The cell growth and the concentration of glucose were constantly monitored during the fermentation process. Glucose was feed into the culture continuously and the speed of the glucose feeding was controlled to keep its concentration in the culture between 0~1 g/L. GGOH (98% pure) was prepared to construct a standard curve for determining GGOH production.

### GGOH analysis by GC-TOF/MS

The GGOH analysis was carried out by GC-TOF/MS according to the previous studies^5,38^. The dodecane layer was sampled and diluted in hexane. 1 μL sample was injected by Agilent 7683 autosampler into Agilent 6890 GC, which was equipped with a fused silica capillary column (30 m × 0.25 mm i.d., 0.25 mm DB-5MS, J&W Scientific, Folsom, CA). The injector temperature was set at 260 °C. The column effluent was introduced into the ion source (250 °C) of TOF/MS. And ions were generated by 40 mA ionization current of a 70 eV electron beam. The mass scan range was 50–800 m/z.

For GC-TOF/MS analysis of GGOH, the oven temperature was first kept constant at 70 °C for 1 min, then increased to 115 °C at a rate of 15 °C/min and kept for 1 min. Next, it increased to 250 °C at a rate of 20 °C/min, and finally increased to 300 °C at a rate of 25 °C/min, kept for 7 min. The total run time was 20.75 min. The GGOH was identified by the mass fragments 69, 93, and 119 m/z.

### Real-time PCR for transcriptional analysis and copy numbers verification

Transcriptional expression levels of the genes *BTS1-ERG20*, *GGPPSsa*, and *tHMGR* in each constructed strain with different GGOH productions were evaluated by qPCR. The extraction of total RNA was performed using Trizol solution (Invitrogen). The PCR procedure was carried out on a CFX96 real time PCR system (Bio-Rad) in a total volume of 20 μL containing diluted cDNA (1 μL), 2 × SsoFast supermix (10 μL), 10 μM forward primer and reverse primer (0.8 μL for each) and 50 × ROX Reference Dye II (0.4 μL). The cycling condition used was 95 °C for 2 min, followed by 40 cycles of 95 °C for 10 s and 58 °C for 20 s.

δ-integration method can obtain a various range of copy numbers. Thus, it’s necessary to verify the copy numbers of each engineered strain by quantitative PCR, which can uncover the relationship between the copy numbers and yields. As the three genes *BTS1-ERG20*, *GGPPSsa* and *tHMGR* were integrated sequentially into the yeast chromosome, their copy numbers were the same theoretically except the endogenous ones. Thus, only the gene *GGPPSsa* of each constructed strain with different GGOH productions were evaluated by qPCR. *ALG9* gene in the chromosome was chosen as the reference gene that was verified with one copy in the strain genome.

## Electronic supplementary material

Supplementary Information
