# Supplementary material for: Engineering Saccharomyces cerevisiae for geranylgeraniol overproduction by combinatorial design
Source: Sci Rep. 2017 Nov 8;7:14991. doi: 10.1038/s41598-017-15005-4 (PMC5678108; doi:10.1038/s41598-017-15005-4)
Supplement: Supplementary file 1 — Supplementary Information [file 41598_2017_15005_MOESM1_ESM.pdf]

Supporting Information

Engineering *Saccharomyces cerevisiae* for geranylgeraniol overproduction by combinatorial design

Tian-Qing Song[a,b] , Ming-Zhu Ding[a,b], Fang Zhai[a,b], Duo Liu[a,b], Hong Liu[a,b],

Wen-Hai Xiao[a,b]* , Ying-Jin Yuan[a,b]

a: Key Laboratory of Systems Bioengineering (Ministry of Education), Tianjin University, Tianjin, 300072, PR China

b: SynBio Research Platform, Collaborative Innovation Center of Chemical Science and Engineering (Tianjin), School of Chemical Engineering and Technology, Tianjin University, Tianjin, 300072, PR China

*Corresponding author: Wen-Hai Xiao (Email: wenhai.xiao@tju.edu.cn, Tel: 86-22-27401288，Postal address: No. 92, Weijin Road, Nankai District, Tianjin, 300072, PR China)

Supplementary Table S1

Integration sites analyzed in 10 strains from 4 combinations with distinct GGOH production.

Strain	GGOH (mg/L)	Integration site
03-1	70.78	XII-2
03-7	124.53	XVI-2
03-8	161.82	XVI-1
04-1	41.22	X-1
04-7	116.26	XVI-2
04-8	145.19	XV-1
09-1	36.32	XII-2
09-8	127.59	X-1
10-1	48.90	X-1
10-8	133.39	XVI-1

Supplementary Table S2

2-level fractional factorial design for determining the notable factors in fermentation medium.

Run	Glucose (g/L)	Yeast extract (g/L)	Peptone (g/L)	$MgSO_4$ (g/L)	KH_2PO_4 (g/L)	GGOH/ (mg/L)
1	50	1	30	0	10	298.96
2	10	1	1	10	0	15.02
3	50	1	1	0	0	28.25
4	50	15	1	10	0	372.85
5	10	15	30	10	0	92.01
6	50	15	1	0	10	180.01
7	50	1	30	10	0	243.28
8	10	1	1	0	10	18.26
9	50	15	30	0	0	360.65
10	10	1	30	0	0	48.94
11	10	1	30	10	10	64.81
12	50	15	30	10	10	418.28
13	10	15	1	10	10	102.87
14	50	1	1	10	10	20.43
15	10	15	30	0	10	62.39
16	10	15	1	0	0	86.17

Supplementary Table S3

RSM with Box-Behnken design for medium optimization.

Run	Glucose (g/L)	Yeast extract(g/L)	Peptone(g/L)	GGOH(mg/L)
1	50.0	15.5	30.5	424.09
2	90.0	30.0	30.5	147.12
3	50.0	1.0	1.0	20.43
4	10.0	15.5	60.0	105.59
5	50.0	30.0	1.0	293.56
6	90.0	1.0	30.5	139.63
7	50.0	15.5	30.5	426.28
8	90.0	15.5	1.0	105.65
9	50.0	1.0	60.0	269.93
10	10.0	1.0	30.5	64.81
11	50.0	30.0	60.0	169.06
12	50.0	15.5	30.5	437.29
13	90.0	15.5	60.0	152.92
14	10.0	15.5	1.0	102.87
15	10.0	30.0	30.5	59.12
16	50.0	15.5	30.5	402.16
17	50.0	15.5	30.5	397.82

Supplementary Table S4

cassettes built in this study.

**Gene Name**	**Cassette Description**
ura3	YPRC15up-*ura3*-CYC1t<br>delta1-*ura3*-CYC1t
ggppssa	CYC1t-TPI1p-*ggppssa* -TEF1t<br>CYC1t- HXT7p-*ggppssa*-TEF1t<br>CYC1t-TDH1p -*ggppssa*-TEF1t<br>CYC1t-TDH3p-*ggppssa*- TEF1t
bts1-erg20	TEF1t-GPM1p-*bts1-erg20*-TPI1t<br>TEF1t- TDH2p -*bts1-erg20*-TPI1t<br>TEF1t- HXK2p -*bts1-erg20*-TPI1t<br>TEF1t- TEF1p -*bts1-erg20*-TPI1t
thmgr	TPI1t-PDC1p-*thmgr*-FBA1t- YPRC15dn<br>TPI1t-PDC1p-*thmgr*-FBA1t- delta2<br>TPI1t- PGK1p -*thmgr*-FBA1t- delta2<br>TPI1t- PGI1p -*thmgr*-FBA1t- delta2<br>TPI1t- TEF1p -*thmgr*-FBA1t- delta2

Supplementary Table S5

Primers used in this study.

Name	Sequence (5'-3')
**Primers for gene amplification in the gene cassettes construction step. The primers pair "gene name-promoter name-F" and "gene name-terminator name-R" were used for the Golden Gate restriction-ligation method, and the enzyme sites were marked with underline. And the primers pair "gene name-promoter name-F -Y" and "gene name-terminator name-R-Y" were used for yeast homologous recombination method.**	
GGPPSsa-TPI1p-F/ GGPPSsa-HXT7p-F	GGTCTCCAAAAATGTCTTACTTCGACAACTACTTTAACG
GGPPSsa-TDH1p-F/ GGPPSsa-TDH3p-F	GGTCTCCCAAAATGTCTTACTTCGACAACTACTTTAACG
GGPPSsa-TEF1t-R	GGTCTCCATTTTTATTTCCTTCTTCTAATGGTAAACTCTG
BE-GPM1p-F	GGTCTCCAATAATGGAGGCCAAGATAGATGAGC
BE-TDH2p-F	GGTCTCCCAAAATGGAGGCCAAGATAGATGAGC
BE-HXK2p-F	GGTCTCCTAAAATGGAGGCCAAGATAGATGAGC
BE-TPI1t-R	GGTCTCCAATCCTATTTGCTTCTCTTGTAAACTTTGTTC
BE-TEF1p-F-Y	GAAAGCATAGCAATCTAATCTAAGTTTTAATTACAAAATGGAGG CCAAGATAGATGAGC
BE-TPI1t-R-Y	GAAGATAATATTTTTATATAATTATATTAATCCTATTTGCTTCTCTT GTAAACTTTGTTC
tHMGR-PDC1p-F	GGTCTCCCAAAATGGACCAATTGGTGAAAACTG
tHMGR-PGK1p-F	GGTCTCCAACAATGGACCAATTGGTGAAAACTG
tHMGR-PGI1p-F	GGTCTCCAAAAATGGACCAATTGGTGAAAACTG
tHMGR-FBA1t-R	GGTCTCCTAACTTAGGATTTAATGCAGGTGACGG
tHMGR-TEF1p-F-Y	GAAAGCATAGCAATCTAATCTAAGTTTTAATTACAAAATGGACC AATTGGTGAAAACTG
tHMGR-FBA1t-R-Y	CTCATTAAAAAACTATATCAATTAATTTGAATTAACTTAGGATTTA ATGCAGGTGACGG
**Primers for amplification of the promoters / terminators and OE-PCR of the related fragments. A pair of primers "terminator-promoter-F" and "promoter-terminator-R" were used for amplification of the promoter fragment while the primers pair "promoter-terminator-F" and "terminator-promoter-R" were for amplification of the terminator fragment. For example, the primer pair "GPDt-PDC1p-F" and "PDC1p-FBA1t-R" were for amplification of the promoter PDC1p, which was subsequently to construct the empty cassette GPDt-PDC1p- FBA1t by OE-PCR.**	
**CYC1t- (TPI1p/ HXT7p/ TDH1p/ TDH3p)- TEF1t**	
CYC1t-F	AAAACTGCAGGCGGCCGCCATGTAATTAGTTATGTCACGCTTAC
CYC1t-TPI1p-R	CAACCTGATGGGTTCCTAGATATAAAAGCCTTCGAGCGTCCC

CYC1t-HXT7p-R	CGAAATTGTTCCTACGAGAAGTAAAGCCTTCGAGCGTCCC
CYC1t-TDH1p-R	GTGATATAGAGTGTAAATGAGCATATACAAAAGCCTTCGAGCGTCCC
CYC1t-TDH3p-R	AAATGGCAGTATTGATAATGATAAACTAAAGCCTTCGAGCGTCCC
CYC1t-TPI1p-F	GGGACGCTCGAAGGCTTTTATATCTAGGAACCCATCAGGTTG
TPI1p-TEF1t-R	GAAAAGTCTTATCAATCTCCTTATTTGGAGACCGGTCTCCTTTTAGTTTATGTATGTGTTTTTTGTAGT
CYC1t-HXT7p-F	GGGACGCTCGAAGGCTTTACTTCTCGTAGGAACAATTTCG
HXT7p-TEF1t-R	GAAAAGTCTTATCAATCTCCTTATTTGGAGACCGGTCTCCTTTTTGATTAAAATTAAAAAAACTTTTTG
CYC1t-TDH1p-F	GGGACGCTCGAAGGCTTTTGTATATGCTCATTTACACTCTATATCAC
TDH1p-TEF1t-R	GAAAAGTCTTATCAATCTCCTTATTTGGAGACCGGTCTCCTTTGTTTTGTGTGTAAATTTAGTGAAG
CYC1t-TDH3p-F	GGGACGCTCGAAGGCTTTAGTTTATCATTATCAATACTGCCATTT
TDH3p-TEF1t-R	GAAAAGTCTTATCAATCTCCTTATTTGGAGACCGGTCTCCTTTGTTTGTTTATGTGTGTTTATTCG
TPI1p-TEF1t-F	ACTACAAAAAACACATACATAAACTAAAAGGAGACCGGTCTCCAAATAAGGAGATTGATAAGACTTTTC
HXT7p-TEF1t-F	CAAAAAGTTTTTTTAATTTTAATCAAAAAGGAGACCGGTCTCCAAATAAGGAGATTGATAAGACTTTTC
TDH1p-TEF1t-F	CTTCACTAAATTTACACACAAAACAAAGGAGACCGGTCTCCAAATAAGGAGATTGATAAGACTTTTC
TDH3p-TEF1t-F	CGAATAAACACACATAAACAAACAAAGGAGACCGGTCTCCAAATAAGGAGATTGATAAGACTTTTC
TEF1t-R	CGCGGATCCGCGGCCGCGATAGCGCCGATCAAAGTA
**TEF1t-(GPM1p/ TDH2p/ HXK2p/ TEF1p) -TPI1t**	
TEF1t-F	AAAACTGCAGGCGGCCGCAAATAAGGAGATTGATAAGACTTTTC
TEF1t-GPM1p-R	CTTAAAGTCATACATTGCACGACTAGATAGCGCCGATCAAAGTA
TEF1t-TDH2p-R	TCATTCACTGGAAAAACCAATGATAGCGCCGATCAAAGTA
TEF1t-HXK2p-R	TGCTCTTCTATGGCGTTCAGATAGCGCCGATCAAAGTA
TEF1t-TEF1p-R	GAAGAGTAAAAAAGGAGTAGAAACATTGATAGCGCCGATCAAAGTA
TEF1t-GPM1p-F	TACTTTGATCGGCGCTATCTAGTCGTGCAATGTATGACTTTAAG
GPM1p-TPI1t-R	GAAGATAATATTTTTATATAATTATATTAATCTGAGACCGGTCTCCTATTGTAATATGTGTGTTTGTTTGG
TEF1t-TDH2p-F	TACTTTGATCGGCGCTATCATTGGTTTTTCCAGTGAATGA
TDH2p-TPI1t-R	GAAGATAATATTTTTATATAATTATATTAATCGGAGACCGGTCTCATTTGTTTTGTTTGTTTGTGTGATG
TEF1t-HXK2p-F	TACTTTGATCGGCGCTATCTGAACGCCATAGAAGAGCA

HXK2p-TPI1t-R	GAAGATAATATTTTTATATAATTATATTAATCTGAGACCGGTCTCCTTTATTTAATTAGCGTACTTATTATGTG
TEF1t-TEF1p-F	TACTTTGATCGGCGCTATCAATGTTTCTACTCCTTTTTACTCTTC
TEF1p-TPI1t-R	TATAATTATATTAATCGTTTAAACTTTGTAATTAAAACTTAGATTAGATTGCTATG
GPM1p-TPI1t-F	CCAAACAAACACACATATTACAATAGGAGACCGGTCTCAGATTAATATAATTATATAAAAATATTATCTTC
TDH2p-TPI1t-F	CATCACACAAACAAACAAAACAAATGAGACCGGTCTCCGATTAATATAATTATATAAAAATATTATCTTC
HXK2p-TPI1t-F	CACATAATAAGTACGCTAATTAAATAAAGGAGACCGGTCTCAGATTAATATAATTATATAAAAATATTATCTTC
TEF1p-TPI1t-F	TAAGTTTTAATTACAAAGTTTAAACGATTAATATAATTATATAAAAATATTATCTTC
TPI1t-R	CGCGGATCCGCGGCCGCTATATAACAGTTGAAATTTGGATAAGAAC
**TPI1t-(PDC1p/ PGK1p/ PGI1p/ TEF1p) -FBA1t- delta2**	
TPI1t-F	AAAACTGCAGGCGGCCGCGATTAATATAATTATATAAAAATATTATC
TPI1t-PDC1p-R	ATGCTCACCCAGTCGCATGTATATAACAGTTGAAATTTGGATAAGAAC
TPI1t-PGK1p-R	TTGAAGTCAGGAATCTAAAATATATATAACAGTTGAAATTTGGATAAGAAC
TPI1t-PGI1p-R	CAATACACCCACACCCACCTATATAACAGTTGAAATTTGGATAAGAAC
TPI1t-TEF1p-R	GAAGAGTAAAAAAGGAGTAGAAACATTTATATAACAGTTGAAATTTGGATAAGAAC
TPI1t-PDC1p-F	GTTCTTATCCAAATTTCAACTGTTATATACATGCGACTGGGTGAGCAT
PDC1p-FBA1t-R	CATTAAAAAACTATATCAATTAATTTGAATTAACGGAGACCGGTCTCCTTTGATTGATTTGACTGTGTTATTTTG
TPI1t-PGK1p-F	GTTCTTATCCAAATTTCAACTGTTATATATATTTTAGATTCCTGACTTCAA
PGK1p-FBA1t-R	CATTAAAAAACTATATCAATTAATTTGAATTAACGGAGACCGGTCTCCTGTTTTATATTTGTTGTAAAAAGTAGATAATTAC
TPI1t-PGI1p-F	GTTCTTATCCAAATTTCAACTGTTATATAGGTGGGTGTGGGTGTATTG
PGI1p-FBA1t-R	CATTAAAAAACTATATCAATTAATTTGAATTAACGGAGACCGGTCTCCTTTTAGGCTGGTATCTTGATTCT
TPI1t-TEF1p-F	GTTCTTATCCAAATTTCAACTGTTATATAAATGTTTCTACTCCTTTTTTACTCTTC
TEF1p-FBA1t-R	ATTAATTTGAATTAACGTTTAAACTTTGTAATTAAAACTTAGATTAGATTGCTATG

PDC1p-FBA1t-F	CAAAATAACACAGTCAAATCAATCAAAGGAGACCGGTCTCCGTTAATTCAAATTAATTGATATAGTTTTTTAATG
PGK1p-FBA1t-F	GTAATTATCTACTTTTTACAACAAATATAAAACAGGAGACCGGTCTCCGTTAATTCAAATTAATTGATATAGTTTTTTAATG
PGI1p-FBA1t-F	AGAATCAAGATACCAGCCTAAAAGGAGACCGGTCTCCGTTAATTCAAATTAATTGATATAGTTTTTTAATG
TEF1p-FBA1t-F	TAAGTTTTAATTACAAAGTTTAAACGTTAATTCAAATTAATTGATATAGTTTTTTAATG
FBA1t- Delta2-R	CACCTGCTTCATCAGCTGTTAAAGATGAGCTAGGCTTTTGTAAAAATATC
FBA1t- Delta2-F	GATATTTTTACAAAAGCCTAGCTCATCTTTAACAGCTGATGAAGCAGGTG
Delta2-R	CGCGGATCCGCGGCCGCGAGAACTTCTAGTATATTCTGTATACCTAATAT
**Primers for verification of the gene insert and the geranylgeraniol pathway integration.**	
TPI1p-seq-F	GCAGCATAATTTAGGAGTTTAGTG
HXT7p-seq-F	TATGCCAATACTTCACAATGTTCG
TDH1p-seq-F	TGGTGATAATGACCAAAC
TDH3p-seq-F	AGTTCCCTGAAATTATTCCCCTAC
TEF1p-seq-F	CTCTTTCGATGACCTCCCATTG
GPM1p-seq-F	CTGTTGCCAGGGAGGGTG
TDH2p-seq-F	TAGTACCCAGTGATCGCAGAC
HXK2p-seq-F	GCACCTTCGCCACTGTC
PDC1p-seq-F	CTCTTTCACTCTCCTTGCAATCAGAT
PGK1p-seq-F	GCATAAATTGGTCAATGC
PGI1p-seq-F	TACTTGCATTCAGTGAATTTTAATAC
TEF1t-seq-R	CATTTGGACTGTCGCCTGTT
FBA1t-seq-R	CTTCAGAAGAAAAGAGCCGACC
PGK1t-seq-R	AGCGTAAAGGATGGGGAAAG
CYC1t-seq-R	TCCTTCCTTTTCGGTTAGAGC
**Primers for verification of the integration site on the yeast chromosome. The format of the reverse primer names were "chromosome-δ site number-length of amplicon-R". The numbers in the parentheses represented the percentage of similarity of the corresponding chromosome δ site sequence with the sequence we used for integration.**	
thmgr-Delta-F	GGTCCGTCACCTGCATTAAATCC
I-1-1590-R (98%)	CAACTACTTAGTCGTGATACAACAGTATGGATAG
IV-1-1382-R (95%)	CAATCGTCCTCTCTTGGCATAGTAGACACAG
IV-2-1650-R (94%)	TATTGACTGAACGAATGCTGAGTAGGCGAGG
IV-3-1488-R (92%)	TACTGTGATTTTGAATACACTGGAATAGGGG
IV-4-1527-R (92%)	GTTTGAAAGGTCTTTGGCACAGAAACTTCG

IV-5-1432-R (91%)	GATAGGTAACTCTTGTGAATAAGTGTTGGG
V-1-1713-R (98%)	CTGTTAGTCCATCAATATACGCCATCTTTT
V-2-1590-R (91%)	TCCCGAGAAATACTACAACGACAGTGATAC
VII-1-1396-R (93%)	GCTAAATAATCTAAAGGGAAACAGCCCAAAAC
VII-2-1545-R (93%)	TCTCAACATTCCCCCATTTTTCAAAGGTAATT
X-1-1397-R (94%)	TGCTGATGTGATGACAAAACCTCTTCCGAT
X-2-1532-R (93%)	CGAGTAAATACGCCTCCTTTTTATCTGTGAATGAC
XII-1-1543-R (98%)	CACCCACTACACTACTCGGTCAGGCTCTTAC
XII-2-1570-R (94%)	CATTATCTTATTACATTATCAATCCTTCCATTTCAGC
XII-3-1473-R (94%)	GTATAAACCTGACTTAGAAGACTTTTTGATGC
XII-4-1592-R (91%)	CGTATTAATTGTTGGGATTTCGTTGTTGATATTAGGT
XIII-1-1447-R (98%)	CTTAGCCACTAAGGAATCACCGAATAGTAAAAG
XIII-2-1457-R (91%)	GCGAACAATAGAATCCCAACGGTTATCAAAT
XIV-1-1510-R (94%)	CAACTCGTGCTCTGCTGGCTATAAAAGGGGT
XIV-2-1539-R (91%)	CGATTAACAGTCGCACGCCTTAACCAACTT
XV-1-1481-R (92%)	TGTCAAAATAGCCGCCCAACTTCTACGCCC
XVI-1-1785-R (98%)	TGGACAGTTGTTACAGTTGCCCGTGAAGGT
XVI-2-1398-R (95%)	AACCGCTTCCACATCTGAATCATATCTCCG
XVI-3-1805-R (93%)	GAAAATCACTAAGCCACAAAAACAATAAAG
XVI-4-1653-R (91%)	GTCACCCTGTAAGGCTTTCTACTCTATCAC

Supplementary Fig. S1. GC-MS results for GGOH standard, host cell BY4742, and SYBE_Sc01010369.

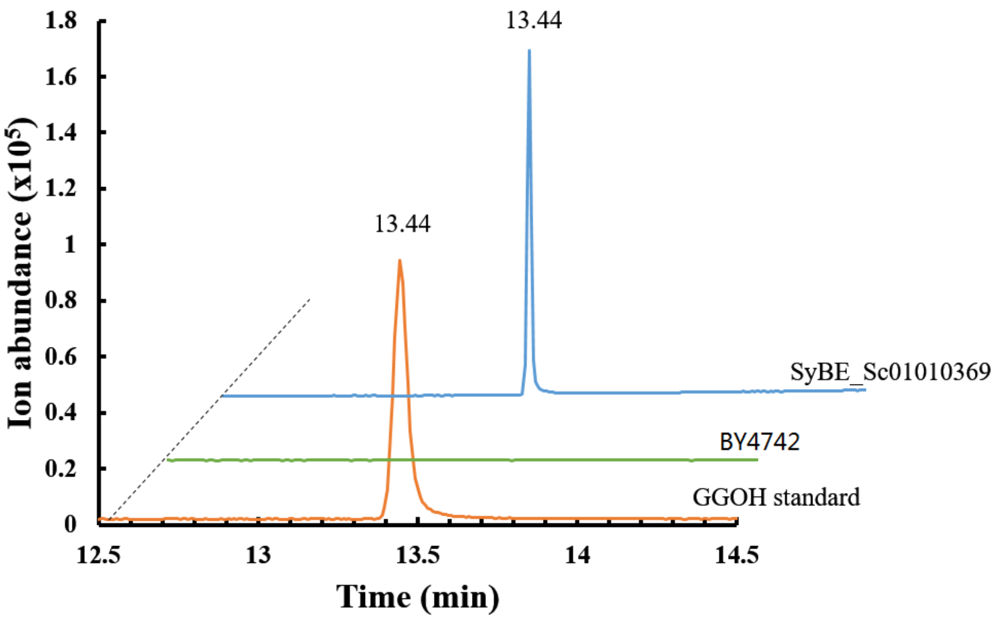

Supplementary Fig. S2. Promoters strength characterization in our study. X-axis showed the mean fluorescence intensity of RFP while Y-axis listed the names of the promoters. The experiments were carried out in 96 well plate. Error bars represented the standard deviations from three independent experiments. The control was wild strain without RFP expression.

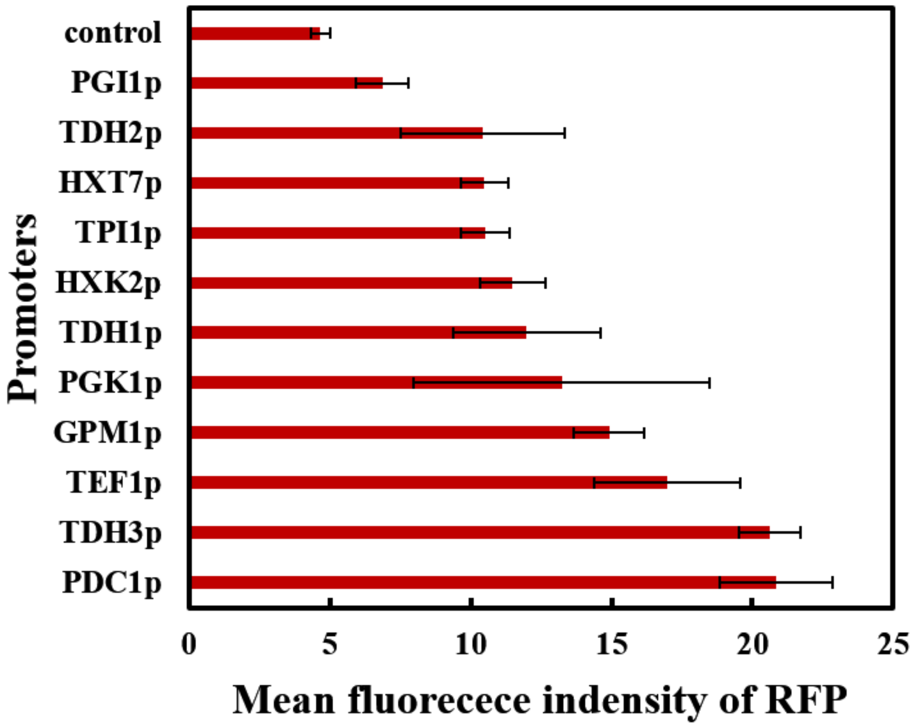

Supplementary Fig. S3. Integration site detection. (a) Schematic diagrams of the integration sites detection. The genomic DNA of the chassis BY4742 was used as the negative control. (b) 6 verified integration sites by PCR.

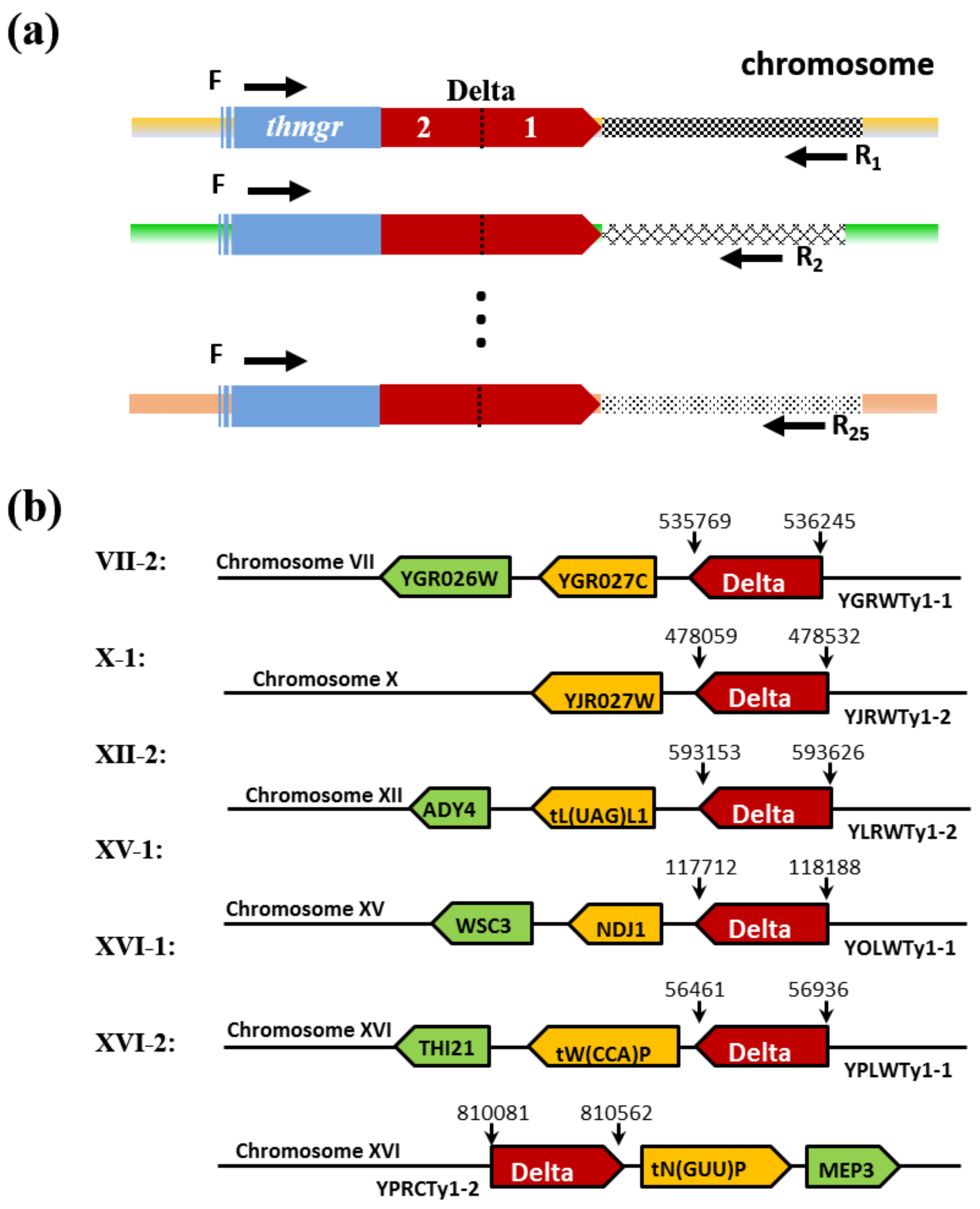

Supplementary Fig. S4. Half-normal probability plot of the results of the 2-level fractional factorial design. The factors glucose (A), yeast extract (B) and peptone (C) displayed far off the near zero line (red line) on the plot were considered to be the notable ones, while $MgSO_4$ and KH_2PO_4 which were not marked were displayed on or close to the near zero line and were defined as unimportant factors.

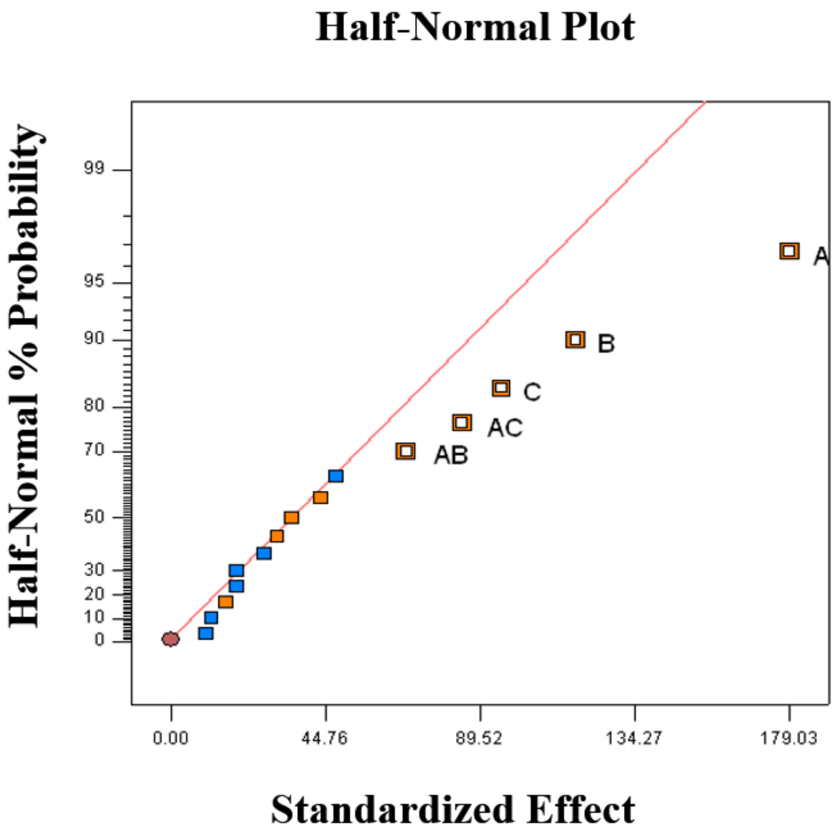

Supplementary Equation S1. The fitted equation (in terms of coded values) for the prediction of geranylgeraniol production (mg/L). A, B and C stand for glucose, yeast extract and peptone, respectively.

Geranylgeraniol = 417.53 + 26.62 * A + 21.76 * B + 21.87 * C + 3.29 * A * B + 11.14 * A * C - 93.5 * B * C - 193.17 * A^2 - 121.69 * B^2 - 107.60 * C^2
